# Evaluation of Reliability and Validity of Three Common Dry Eye Questionnaires in Chinese

**DOI:** 10.1155/2018/2401213

**Published:** 2018-08-27

**Authors:** Fan Lu, Aizhu Tao, Yinu Hu, Weiwei Tao, Ping Lu

**Affiliations:** ^1^School of Ophthalmology and Optometry, Wenzhou Medical University, Wenzhou, Zhejiang, China; ^2^The First Affiliated Hospital of Wenzhou Medical University, Wenzhou, Zhejiang, China

## Abstract

**Purpose:**

To investigate the psychometric properties of three commonly used dry eye questionnaires including McMonnies Questionnaire (MQ), the Ocular Surface Disease Index (OSDI), and the Salisbury Eye Evaluation Questionnaire (SEEQ) in Chinese.

**Methods:**

This prospective cross-sectional study was conducted at the Eye Hospital of Wenzhou Medical University. Ninety-eight participants completed three questionnaires in a random order. Ophthalmic examinations including tear break-up time, corneal fluorescein staining score, and Schirmer I test were performed. Reliability, validity, and accuracy were assessed for three questionnaires.

**Results:**

There were 35 mild-to-moderate dry eye patients, 14 severe dry eye patients, and 49 non-dry eye patients. The Cronbach *α* of MQ, OSDI, and SEEQ was 0.54, 0.74, and 0.76, respectively, and the intraclass correlation coefficients were 0.91, 0.90, and 0.94, respectively. There were significant differences (*P* < 0.05) in MQ and OSDI scores among different groups, but there were no statistically significant differences between the mild-to-moderate group and the severe group in terms of SEEQ scores. With cutoff values for abnormal MQ of 15, OSDI of 27.2, and SEED of 1, respectively, good dry eye diagnostic accuracies were obtained.

**Conclusions:**

The three questionnaires showed fair accuracy in the diagnosis of dry eye. The cutoff values of OSDI changed when applied to Chinese people.

## 1. Introduction

Dry eye is a kind of disease caused by abnormal tear quantity and quality or decreased stability of tear film due to abnormal tear dynamics, and it is accompanied by eye discomfort and/or tissue lesions characteristics of the eye [1]. Dry eye symptoms may be present in the absence of significant damage to the ocular surface, and one of the goals of dry eye treatment is to improve symptoms. Therefore, it is of great significance to evaluate the symptoms in the diagnosis and monitoring of the therapeutic efficacy of dry eye.

As early as 1986, McMonnies [2] pointed out that surveying a patient's medical history is of great significance for dry eye diagnosis and designed McMonnies dry eye questionnaire (McMonnies Questionnaire (MQ)). Since then, a series of dry eye questionnaires has been developed for epidemiological investigation and clinical study of dry eye [3, 4]. At present, the dry eye questionnaire used in clinical or scientific research in China is mainly based on the direct translation of foreign questionnaires. Three kinds of questionnaires have been used widely, including MQ, the Ocular Surface Disease Index (OSDI), and the Salisbury Eye Evaluation Questionnaire (SEEQ). These questionnaires differ in length and design, and there is a certain degree of blindness in the choice of questionnaire in clinical or scientific research. As the research object of these questionnaires is Western, when it is applied to the Chinese people, its reliability and validity will be changed, and the diagnostic value of dry eye will also need to be reevaluated. Therefore, in the present study, we compared the psychometric characteristics (reliability, validity, and accuracy) of the three common dry eye questionnaires, providing information for the further design and improvement of dry eye questionnaires.

## 2. Subjects and Methods

The study was approved by the human subjects' review board at the Eye Hospital of Wenzhou Medical University. Each subject signed a consent form and was treated in accordance with the principles of the Declaration of Helsinki. Ninety-eight subjects (46 females and 52 males, mean age: 33.4 ± 16.8 year, ranging from 18 to 76 years) were recruited from either students at Wenzhou Medical University or outpatients at the Affiliated Eye Hospital of Wenzhou Medical University. Patients with systemic or other ocular diseases were excluded from this study.

All subjects first completed the MQ, OSDI, and SEEQ in a random order and then had objective eye examinations according to the following order: tear film break-up time (TBUT), fluorescein corneal staining (FL), and Schirmer I test (SIT). Tear break-up time was defined as the interval between the last complete blink and the appearance of the first black spot. The mean value of 3 measurements of each eye was calculated. The cornea of each eye was divided into upper, middle, and lower parts during fluorescein staining score evaluation, with each part being graded from 0 to 3: no staining was defined as grade 0, tiny and scattered dyeing was grade 1, larger and diffuse staining was grade 3, staining between 1 and 3 was defined as 2, and the total score ranged from 0 to 9 points [5]. The length of wetting of filter strip in 5 minutes after surface anesthesia was measured and recorded as the Schirmer test value.

2-3 weeks after the first questionnaire survey, a survey of three dry questionnaires was derived via telephone interview. If subjects had dry eye intervention (such as the use of artificial tears, tears embolism, etc.) or ocular surgery or other eye diseases during the two surveys, the subjects were not included in the repeatability analysis.

The Japanese diagnostic criterion of dry eye was adopted in the present study [6]: (1) subjective symptoms of dry eye; (2) TBUT < 5 s or SIT < 5 mm/5 min; (3) ocular surface lesions: corneal fluorescein staining was greater than 3 points. If all criteria mentioned above were positive, the subject was diagnosed as dry eye. To evaluate the severity of dry eye, the following grade standard was adopted [7]: (1) at least three dry eye symptoms often occur; (2) corneal fluorescein staining was greater than 6; (3) TBUT < 5 s; (4) SIT < 5 mm/5 min. Any of the above being positive was recorded as 2 points, whereas negative results were recorded as 1 point. The scores of 4 items were added together, and a total score of 4–6 points were identified as mild-to-moderate dry eye, whereas 7-8 points was identified as severe dry eye.

The SPSS Statistical Package Program 13.0 (SPSS, Cary, NC) was used for data processing; *P* < 0.05 was considered statistically significant. Data are presented as the mean ± standard deviations (SD). Reliability refers to the consistency of a measuring test. The internal consistency reliability of each questionnaire was evaluated by Cronbach's alpha coefficient; the test-retest reliability of each questionnaire was tested by the intraclass correlation coefficient (ICC). Validity refers to the degree to which a measurement corresponds accurately with reality. The construct validity of the questionnaire was evaluated with factor analysis, the concurrent validity of each questionnaire was assessed by Spearman correlation analysis, and the discriminant validity of each questionnaire was evaluated by using the multiple comparison of rank sum test. Accuracy is the degree of closeness of a measurement to its actual value. The accuracy of each dry eye questionnaire for the diagnosis of dry eye was described by the area under the receiver operating characteristic curve (ROC). Based upon the normal MQ of 12.82 ± 5.21 [8], the minimum sample size to detect a 9.0 group difference with a 99% statistical power was 28 [9]. Similarly, based upon the normal OSDI of 9.6 ± 14.2 [7], the minimum sample size to detect a 20.0 group difference with a 99% statistical power was 40 [9]. Therefore, the 98 cases in this study were more than adequate.

## 3. Results

Ninety-eight subjects were recruited for this study: among these, 49 cases were diagnosed with dry eye (mild-to-moderate dry eye in 35 cases and severe dry eye in 14 cases). Another investigation was performed 2-3 weeks after the first survey: subjects who had either drug or surgical intervention for dry eye were removed, and 39 measurements were obtained for repeatability analysis. The average age of the dry eye group was 35.3 ± 18.1 years, the average age of the non-dry eye group was 31.4 ± 15.2 year, and there was no significant difference in age and gender between the two groups (*P*=0.564  and  0.544, resp., *χ*^2^ test). Dry eye questionnaire scores and clinical dry eye test results were statistically significantly different between the two groups (*P* < 0.001, Wilcoxon rank sum test, Table 1).

### 3.1. Reliability

The Cronbach's alpha coefficient of MQ was 0.54, and the average correlation coefficient of each item was 0.22, indicating that the internal consistency reliability of the questionnaire was low. The overall alpha reliability coefficient of OSDI was 0.74, and the alpha reliability coefficients of “eye symptoms,” “visual function,” and “environmental triggering factors” were 0.67, 0.71, and 0.86, respectively, indicating that the internal consistency reliability of the scale was relatively high. The overall alpha reliability coefficient of SEEQ was 0.76, and the average correlation coefficient of each item was 0.50, showing that the internal consistency was good.

The MQ scores of the two surveys were 10.2 ± 6.5 and 10.3 ± 6.3, the OSDI scores were 22.7 ± 13 and 22.5 ± 10.5, and the SEEQ scores were 0.6 ± 1 and 0.7 ± 0.9. Their ICC were 0.91 (95% confidence interval: 0.87–0.94), 0.90 (95% confidence interval: 0.85–0.94), and 0.94 (95% confidence interval: 0.90–0.96), respectively, showing that the test-retest reliability of the questionnaires was good.

### 3.2. Validity

The construct validity of the questionnaire was evaluated by factor analysis. The scores of each item in the questionnaire were rotated with the maximum variance, and the principal component analysis method was used for analysis. MQ and OSDI each identified 4 main common factors, and SEEQ identified 2 common factors (characteristic value was greater than 1). The factor loads of each item of the questionnaire were only on one factor and were in middle to high degree (>0.4, Tables 2–4), suggesting that the factors are independent of each other and the questionnaires have good construct validity.

Relationships between the questionnaires and the objective examination results of the dry eye group are shown in Table 5. We found that MQ and OSDI scores were positively correlated (*r*=0.597, *P* < 0.001), the MQ score was positively correlated with the SEEQ score (*r*=0.381, *P*=0.01), and the OSDI score was positively correlated with the SEEQ score (*r*=0.400, *P*=0.01). Apart from the MQ score being negatively correlated with SIT values (*r*=−0.309, *P*=0.03) and the SEEQ score being positively correlated with the corneal fluorescein staining (*r*=360, *P*=0.01), the questionnaire scores and other objective eye examination results were not correlated (*P* > 0.05).

There was significant difference in the MQ scores between the non-dry eye group and both the mild-to-moderate dry eye group and the severe dry eye group (*P* < 0.001, Table 6). Furthermore, the MQ score of the mild-to-moderate dry eye group was significantly different from that of the severe dry eye group (*P* < 0.001, Table 6). The OSDI total score and the dimension “visual function” were statistically significant among different groups (*P* < 0.01 for all comparisons, Table 6). There was statistical difference between the non-dry eye group and the mild-to-moderate dry eye group in the dimension “eye symptom” (*P* < 0.001, Table 6); there was no significant difference between the non-dry eye group and the mild-to-moderate dry eye group in the dimension “environmental trigger” (*P*=0.04, *α* = 0.017, according to *α* = 0.05; the number of comparisons, Table 6); there was no statistically significant difference in the SEEQ score between the mild-to-moderate dry eye group and the severe dry eye group in the dimension of “eye symptoms”; and “environmental trigger” and SEEQ scores were not statistically different (*P* > 0.02, *α* = 0.017 according to *α* = 0.05; comparison of the number of times, Table 6), although the SEEQ scores among other groups were statistically significantly different (*P* < 0.02, *α* = 0.017 according to *α* = 0.05; comparison of the number of times, Table 6).

### 3.3. Accuracy

**Figure 1:**
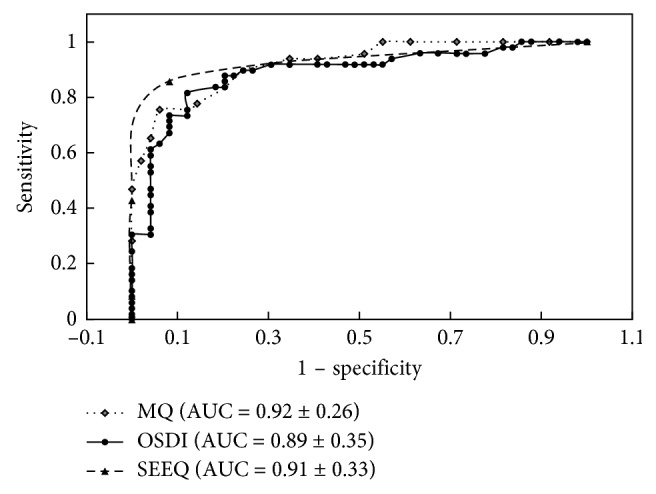
Receiver operating characteristic (ROC) curve of MQ, OSDI, and SEEQ. The area under the ROC curve (AUC) ranged from 0.89 to 0.92. MQ had the largest area, 0.92. When the cutoff value for abnormal MQ was 15, good diagnostic accuracy was obtained with 75.5% sensitivity and 93.9% specificity. MQ: McMonnies Questionnaire; OSDI: Ocular Surface Disease Index; SEEQ: Salisbury Eye Evaluation Questionnaire.

Using sensitivity as the longitudinal coordinate and 1-specificity as the horizontal coordinate, the ROC curves of MQ, OSDI, and SEEQ were constructed (Figure 1). The areas under the ROC curve were 0.92 ± 0.26, 0.89 ± 0.35, and 0.91 ± 0.33, respectively, showing that the values of the three questionnaires in the diagnosis of dry eye were high, especially for the MQ. The diagnosis threshold is determined when the sum of sensitivity and specificity is largest in this diagram. The diagnostic threshold value of MQ was 14.5 (as the questionnaire score is an integer, the actual diagnostic threshold value was 15); the OSDI diagnostic threshold was 27.2; and the diagnostic threshold value of SEEQ was 1. With the cutoff values mentioned above, the sensitivity and specificity of MQ, OSDI, and SEEQ for the diagnosis of dry eye were 75.5% and 93.9%, 75.5% and 87.8%, and 85.7% and 91.8%, respectively.

## 4. Discussions

Dry eye is a chronic, symptomatic ocular surface disease, with symptoms that differ in severity in different patients, and the irritation symptoms of dry eye cause adverse effects on the daily life of patients. However, the presence of a symptom is not always clear, especially when it is hidden, and patients may consider it as an inevitable result of visual symptoms (such as the general embodiment of ageing). The structured design of the questionnaire is helpful for finding these hidden symptoms. The International Dry Eye Workshop (2007) recommends that all clinical trials related to dry eye should include the use of a well-designed and effective questionnaire that evaluates subjective symptoms and visual function. Moreover, the questionnaire may be the best way to determine whether clinical treatment intervention is effective [10].

The importance of medical history in the diagnosis of dry eye was first proposed by McMonnies, who designed the MQ [2]. The total score of MQ is between 0 and 45, with patients whose total score ≥15 points being considered as dry eye [11]. MQ focuses on the risk factors for dry eye and can help to determine both the existence of dry eye and individuals who are exposed to the risk factors for dry eye. However, the recall period is not specified, symptoms that happened a long time ago may be overlaid with current symptoms. Meanwhile, as the symptoms and influencing factors are mixed in the answers, it is difficult to analyze the influencing factors after the diagnosis of dry eye. In the present study, we found that the alpha reliability coefficient of MQ was 0.54, indicating that the internal consistency reliability is low. As MQ covers a wide range of factors, it has a certain “heterogeneity”; this result can also be seen from factor analysis. Low internal consistency also suggests that, when using MQ either to compare two control groups or to conduct a longitudinal study, a large sample is needed. MQ has good reliability and construct validity. As some of the MQ's questions (such as age, gender, previous dry eye treatment, and medication history) did not change during the 2-3 weeks of our follow-up, this partly explained the observed ICC values. Assessment of the discriminant validity showed that MQ was effective at distinguishing the non-dry eye from the dry eye. Moreover, the level of score has a discriminant value for the severity of the dry eye. The higher the score, the more serious the degree of dry eye; these results were inconsistent with the results of Nichols et al. [12].

OSDI was designed by the Allergan research team [3]. The purpose of this questionnaire is to rapidly assess eye irritation symptoms associated with dry eye and the effects of these symptoms on visual function. Due to the subblock answer and the designated recall period of one week, patients can evaluate themselves each week. However, as the questionnaire does not involve dry eye-related factors (such as drug usage, etc.), it does not facilitate the patient's etiological therapy. We assume that the condition of the patient during the two repeated measurements is stable; however, in fact, the state of a typical dry eye patient is often volatile and inevitably affects the outcome of the retest. There were significant differences in the OSDI total score and the scores of “visual function” between the non-dry eye group and the dry eye group and between the mild-to-moderate dry eye group and the severe dry eye group. This showed that OSDI could not only identify non-dry eyes and dry eyes but also gauge the severity of dry eye. There was no significant difference between the scores of the mild-to-moderate dry eye group and the severe dry eye group in the dimensions of “eye symptoms” and “environmental trigger,” indicating that simple eye symptoms and environmental factors have little value in judging the severity of dry eye; this differs from the results of Schiffman et al. [7]. This may be either because some severe dry eye symptoms in this experiment were tolerated or because relative corneal sensation decreased, accompanied by deterioration of the disease.

SEEQ was proposed by Schein et al. [4] and was originally designed for the epidemiological study of dry eye in old people. It involves 6 symptoms and signs of the eye. According to the frequency of occurrence, when at least one of the symptoms was frequent, the subject was considered as dry eye. As the questionnaire is simple and clear, the SEEQ is often used to study the epidemiology of dry eye in large populations; however, it misses dry eye patients with no obvious symptoms. The present study found that the alpha reliability coefficient of SEEQ was 0.76, showing that the internal consistency is good and the retest reliability and construct validity are all good. As regards discrimination validity, the results of the present study indicate that SEEQ can distinguish the non-dry eye from the dry eye, but it has no value in discriminating the severity of dry eye; the higher the score, the higher the possibility of dry eye, but this does not mean that the degree of dry eye is more serious.

From the results of concurrent validity, the questionnaire scores were positively correlated with each other. However, the correlation in our study was not high, which suggests that the properties of some dry eye patients measured by OSDI were not reflected in the MQ questionnaire. Considering the different content and structure of the questionnaires, this correlation can be expected. In the present study, we found that the consistency of dry eye symptoms and clinical examination results were poor. This is similar to the results of previous studies on the correlation between dry eye symptoms and signs. Schein et al. [4] surveyed dry eye symptoms in 2,249 elderly people and found that the SIT value was not correlated with the frequency of symptoms; Nichols et al. [13] found that dryness and foreign body sensation of dry eye patients were not correlated with tear meniscus height, the phenol red thread, the SIT value, and corneal fluorescein staining. The lack of correlation between dry eye questionnaires and clinical examination may be due to the dry eye group containing different types of dry eye patients. When using a subtype of patients, the correlation was better [7]. There is a lack of correlation between self-reported symptoms and dry eye clinical examination, which is also a puzzling and difficult problem encountered in clinical dry eye treatment and research.

It is generally accepted that the MQ score is ≥15, and the SEEQ score is ≥1 for patients with dry eye [4, 11]. According to Schiffman et al. [7], the OSDI diagnostic threshold has been identified as 15. The diagnostic threshold of OSDI obtained in the present study differs from that of Western people, this may be due to differences in diagnostic criteria of dry eye, in addition to ethnic differences. As the three questionnaires were designed according to the Western cultural background and living environment, the threshold value of the diagnosis was changed when they were applied to the East. However, large sample population studies will be needed to confirm the specific threshold in future. Domestic dry eye researchers can combine the characteristics of dry eye questionnaires developed in foreign countries with Chinese people's habits and environment, thereby designing questionnaires that are more suitable for Chinese people. Future questionnaires may be focusing on developing electronic data system for assessing the effect of dry eye on quality of life and for self-monitoring.

In conclusion, the three questionnaires showed fair accuracy in the diagnosis of dry eye. The OSDI and the MQ scores were suitable for grading the severity of dry eye, and they were employed to screen individuals for the diagnosis of dry eye in the clinic. Furthermore, the OSDI has designated a recall period in the questionnaire and may be used to evaluate the effects of treatments. In contrast, the SEEQ did not prove suitable in discriminating between different levels of severity in dry eye patients. The SEEQ can be completed much more quickly than the OSDI and MQ, thus it may be the more convenient option for epidemiological studies. The cutoff values of OSDI changed when applied to Chinese people.

## Figures and Tables

**Table 1:** Results of the questionnaires and clinical dry eye test in dry eye group (*n*=49) and non-dry eye group (*n*=49).

	Dry eye	Non-dry eye	*P*
MQ	16.4 ± 4.5	7.0 ± 4.6	<0.001
OSDI	35.8 ± 13.4	15.3 ± 9.9	<0.001
SEEQ	1.4 ± 0.9	0.1 ± 0.3	<0.001
TBUT (s)	3.2 ± 1.1	7.2 ± 3.3	<0.001
SIT (mm/5 min)	3.8 ± 2.2	7.9 ± 4.4	<0.001
FL	5.3 ± 2.2	0.4 ± 0.8	<0.001

MQ: McMonnies Questionnaire; OSDI: Ocular Surface Disease Index; SEEQ: Salisbury Eye Evaluation Questionnaire; TBUT: tear film break-up time; SIT: Schirmer I test; FL: fluorescein corneal staining.

**Table 2:** Structural validity analysis of McMonnies dry eye questionnaire.

Question number	Common factor 1	Common factor 2	Common factor 3	Common factor 4
1	0.106	0.762	−0.013	−0.076
2	0.596	0.319	−0.322	0.359
3	0.840	0.188	0.024	−0.061
4	0.651	−0.172	0.142	0.117
5	0.387	−0.605	−0.146	0.144
6	0.067	−0.481	−0.567	−0.043
7	0.081	0.154	0.631	−0.242
8	0.190	0.499	0.051	0.047
9	0.138	0.111	0.656	0.390
10	0.193	−0.262	0.063	−0.703
11	0.249	−0.375	0.006	−0.624
12	0.685	0.063	0.338	−0.165

**Table 3:** Structural validity analysis of Ocular Surface Disease Index.

Dimension	Question number	Common factor 1	Common factor 2	Common factor 3	Common factor 4
1	1	0.664	−0.011	0.233	0.418
1	2	0.677	0.199	0.069	0.289
1	3	−0.045	0.040	−0.050	−0.855
2	4	−0.211	0.194	0.755	0.174
2	5	0.193	0.072	0.829	−0.147
2	6	−0.024	0.882	0.159	0.008
2	7	0.066	0.688	0.271	0.076
2	8	0.539	0.481	−0.247	−0.466
2	9	0.112	0.860	−0.075	−0.042
3	10	0.786	−0.105	0.059	−0.066
3	11	0.851	0.138	−0.118	−0.261
3	12	0.906	0.083	−0.114	−0.172

**Table 4:** Structural validity analysis of Salisbury Eye Evaluation Questionnaire.

Question number	Common factor 1	Common factor 2
1	−0.014	−0.900
2	0.364	0.780
3	0.578	0.205
4	0.640	0.538
5	0.860	−0.108
6	0.649	0.274

**Table 5:** Relationships between questionnaires and the objective examination results of dry eye group.

	MQ	OSDI	SEEQ	TBUT	SIT	FL
MQ	—	0.597^*∗*^	0.381^*∗*^	−0.156	−0.309^*∗*^	0.146
OSDI	—	—	0.400^*∗*^	−0.246	−0.246	0.079
SEEQ	—	—	—	−0.192	−0.205	0.360^*∗*^
TBUT	—	—	—	—	−0.104	−0.163
SIT	—	—	—	—	—	0.075

^*∗*^There was a relationship between the two parameters (*P* < 0.05). MQ: McMonnies Questionnaire; OSDI: Ocular Surface Disease Index; SEEQ: Salisbury Eye Evaluation Questionnaire; TBUT: tear film break-up time; SIT: Schirmer I test; FL: fluorescein corneal staining.

**Table 6:** MQ, OSDI, and SEEQ evaluated according to the severity of dry eye.

	Non-dry eye (*n*=49)	Mild-to-moderate dry eye (*n*=35)	Severe dry eye (*n*=14)
MQ	7.0 ± 4.6	15.2 ± 4.5	19.4 ± 2.7
OSDI	15.3 ± 9.9	32.2 ± 12.5	44.9 ± 11.7
Eye symptoms' dimension	10.9 ± 12.4	27.4 ± 18.4	39.3 ± 27.0
Visual function dimension	11.8 ± 11.0	20.2 ± 11.7	31.8 ± 7.8
Environmental trigger dimension	24.8 ± 19.1	36.4 ± 23.9	48.8 ± 18.4
SEEQ	0.1 ± 0.3	1.2 ± 0.8	1.9 ± 0.9

There was no statistically significant difference either between the non-dry eye group and the mild-to-moderate dry eye group in the dimension “environmental trigger” or between the mild to moderate dry eye group and severe dry eye group in the dimension of “eye symptoms,” and “environmental trigger” and SEEQ scores were not statistically different. All other comparisons among different groups exhibited statistically significant differences. MQ: McMonnies Questionnaire; OSDI: Ocular Surface Disease Index; SEEQ: Salisbury Eye Evaluation Questionnaire.

## Data Availability

The datasets used and analyzed during the current study are available from the corresponding author on reasonable request.

## References

[1] Lemp M. A., Baudouin C., Baum J. (2007). The definition and classification of dry eye disease: report of the Definition and Classification Subcommittee of the International Dry Eye Workshop (2007). *Ocular Surface*.

[2] McMonnies C. W. (1986). Key questions in a dry eye history. *Journal of the American Optometric Association*.

[3] McAlinden C., Gao R., Wang Q. (2017). Rasch analysis of three dry eye questionnaires and correlates with objective clinical tests. *Ocular Surface*.

[4] Schein O. D., Tielsch J. M., Munoz B., Bandeen-Roche K., West S. (1997). Relationship between signs and symptoms of dry eye in the elderly. A population-based perspective. *Ophthalmology*.

[5] van Bijsterveld O. P. (1969). Diagnostic tests in the Sicca syndrome. *Archives of Ophthalmology*.

[6] Mizuno Y., Yamada M., Miyake Y., Dry Eye Survey Group of the National Hospital Organization of Japan (2010). Association between clinical diagnostic tests and health-related quality of life surveys in patients with dry eye syndrome. *Japanese Journal of Ophthalmology*.

[7] Schiffman R. M., Christianson M. D., Jacobsen G., Hirsch J. D., Reis B. L. (2000). Reliability and validity of the ocular surface disease index. *Archives of Ophthalmology*.

[8] Guo Y., Peng R., Feng K., Hong J. (2016). Diagnostic performance of McMonnies questionnaire as a screening survey for dry eye: a multicenter analysis. *Journal of Ophthalmology*.

[9] Erdfelder E., Faul F., Buchner A. (1996). GPOWER: a general power analysis program. *Behavior Research Methods Instruments and Computers*.

[10] Lemp M. A. (1995). Report of the national eye institute/industry workshop on clinical trials in dry eyes. *CLAO Journal*.

[11] McMonnies C. W., Ho A., Wakefield D. (1998). Optimum dry eye classification using questionnaire responses. *Advances in Experimental Medicine and Biology*.

[12] Nichols K. K., Nichols J. J., Mitchell G. L. (2004). The reliability and validity of McMonnies dry eye index. *Cornea*.

[13] Nichols K. K., Nichols J. J., Mitchell G. L. (2004). The lack of association between signs and symptoms in patients with dry eye disease. *Cornea*.

